# Tree Resilience Indices of Norway Spruce Provenances Tested in Long-Term Common Garden Experiments in the Romanian Carpathians

**DOI:** 10.3390/plants13162172

**Published:** 2024-08-06

**Authors:** Alin Madalin Alexandru, Georgeta Mihai, Emanuel Stoica, Alexandru Lucian Curtu

**Affiliations:** 1 Department of Forest Genetics and Tree Breeding, “Marin Dracea” National Institute for Research and Development in Forestry, 077190 Voluntari, Romania; georgeta.mihai@icas.ro (G.M.); emanuel.stoica@icas.ro (E.S.); 2 Faculty of Silviculture and Forest Engineering, Transilvania University of Brasov, 500123 Brasov, Romania

**Keywords:** Norway spruce, provenance trials, drought resilience, drought stress, climate change

## Abstract

Provenance trials provide a valuable opportunity to evaluate the impact of extreme events on growth and wood properties. In this study, we have evaluated 81 Norway spruce provenances, tested in three provenance trials established in the Romanian Carpathians in 1972. The response to drought of the Norway spruce provenances has been examined using the following tree resilience indices: resistance, recovery, resilience, and relative resilience. The relationship between climate and growth, the correlations between wood traits, and the coordinates of the origin and tree resilience indices were also analysed. In each provenance trial, there were significant differences between provenances and years regarding wood widths and latewood percentage (LWP). Regarding drought extreme events, the years when they occurred in all three provenance trials were 2000 and 2003. Significant differences between provenances for at least one tree resilience index have been found in all provenance trials, for the year 2000. By using subperiods of 25 years, changes in the relationship between climate and growth have been observed. Several provenances with high radial growth and good resistance and/or recovery have been identified. Provenances that performed better in common garden experiments could be used in assisted migration, even in the proximity of the current natural range.

## 1. Introduction

The 1.5 °C limit of global warming might be reached in the first half of the 2030s [1]. As a result, severe and extreme weather events will become more frequent. Among them, drought and heat stress are considered to have the greatest adverse impact on forest ecosystems. The regions most vulnerable to increasing drought risk will be Southern Europe, with the Mediterranean region as a hotspot, and Southeastern Europe [2,3,4].

Although it is considered that forests have a large potential for climate change mitigation, droughts and other extreme weather events can threaten their growth, productivity, genetic diversity, and distribution [5,6,7,8,9,10]. In Europe, drought events have caused significant impacts on forests in the last decades, as the century passes [11]. Between 1987 and 2016, drought was the cause of excess forest mortality for almost half a million ha [12]. 

In Romania, from 1901, every decade has had from one to four years with extreme droughts/rain events. The number of identified droughts has been increasing since 1981 [13]. Annual precipitation is expected to decrease, particularly in the southeastern part of the country [14]. Analysing the predictions made for Romania for the short term, 2021–2050, and the long-term, 2071–2100 versus 1971–2000, the eastern and northeastern parts will have large changes in winter temperature, and the eastern and southern parts will have larger changes for the other seasons, more than 2 or 4 °C, for RCP4.5 and RCP8.5, respectively. Regarding precipitation, it is expected to increase, more so in winter, in the central part of the country. At the same time, for the summer, a decline is seen in most cases, the most affected being the southeastern and southwestern areas [15]. 

With a distribution area of around 30 million ha, Norway spruce (*Picea abies*) is an economically important coniferous species in Europe. The vulnerability of Norway spruce to drought has been expressed in many studies [16,17]. It is highly sensitive to rising temperatures and water shortfall [18] and to declining water availability, and it has a weak adaptive capacity at lower elevations [19,20]. Drought causes a decrease in its vitality [21], which leads to a lower resistance against bark beetles, especially *Ips typographus* [22]. Therefore, the risk of mortality for Norway spruce and other native coniferous species might be greater with increased temperatures and decreased precipitation amounts, especially for populations that are established outside the natural range [23]. 

In Europe, according to RCP 8.5 projections, the frequency of droughts is projected to increase over the whole of Europe [11]. The Norway spruce might retreat in the Alps, the Carpathians, and Scandinavia above 60° latitude, disappearing completely from the Central European lowlands [24]. In Romania, the suitable area lost by Norway spruce until 2100 is estimated to be 8%, while the gained area is around 2% [25]. Mihai et al. [26] have revealed that the climate envelopes for the main forest species in Romania have already shifted to another ecosystem climate. Norway spruce will expand to higher altitudes but will significantly decrease in frequency and lose its habitat, particularly in the Eastern Carpathians.

Although the climate will change more rapidly than some populations can adapt, forest species hold different adaptive capacities to mitigate the impacts of climate change. Resilience to drought is a trait with environmental and genetic components, and different populations of the same species might respond differently to the same climate [27]. Recent studies have shown that there is a genetic variation in the sensitivity/tolerance of forest species against drought events, both within and among populations [28,29,30,31]. Using genome analyses, significant associations were found between SNPs (single-nucleotide polymorphisms) and traits related to the drought adaptation of Norway spruce [32].

Therefore, an approach to ensure the genetic adaptation of forest species to the negative impacts of drought is to select the most resilient provenances [28,33]. Using seed sources that will be climatically adapted during the rotation cycle will increase the resilience of forest ecosystems and will maintain the services provided by them. Selection of the most suitable provenances, together with assisted migration, can be considered the most effective strategy for the management and conservation of forest genetic resources in the face of environmental change.

Field tests, such as provenance trials, provide a valuable opportunity to evaluate the impact of extreme events on growth and wood properties. By testing different provenances in homogenous conditions, the most valuable and adapted provenances can be selected. Therefore, provenance trials can provide important data concerning intraspecific adaptive capacity and the selection of suitable populations for reforestation programs. Several studies have analysed the intraspecific variation in climate growth of Norway spruce in provenance trials [34,35,36,37,38,39]. However, the trials from the previous studies were not comprised of so many provenances, and those provenances were studied at younger ages, than the trials from our study, which are comprised of 81 provenances that cover well the distribution area of the species in Europe and are at half the rotation age of this species in Romania.

To quantify trees’ resilience to climate change, tree-ring width measurements are frequently used. Ring width is linked with conifers’ wood structure; hence, changes in radial growth patterns will alter forest productivity, wood properties, and timber quality [40]. In the first half of the growing season, large and thin-walled earlywood tracheids are formed that are more efficient in water transport but are also more vulnerable to cavitation. In the second half of the growing season, narrow and thick-walled latewood tracheids are formed, which provide mechanical support [41].

This study aimed to assess the growth response and adaptive capacity of Norway spruce provenances originating from thirteen European countries to extreme drought events which have occurred in this region in the last 49 years. The objectives were to (i) determine the genetic variation of wood traits among the Norway spruce provenances, (ii) evaluate the response to drought of the provenances, (iii) determine the relationship between growth and the climate of the trial sites, (iv) compute correlations between wood traits, tree resilience indices, and the geographical coordinates of the provenances’ origin, and (v) provide information for the implementation of sustainable forest management and forest genetic resource conservation.

This study will improve knowledge regarding the adaptive capacity of Norway spruce populations of different geographical origins to the cumulative effects of drought and heat stress associated with climate change.

## 2. Materials and Methods

### 2.1. The Provenance Trials

**Figure 1:**
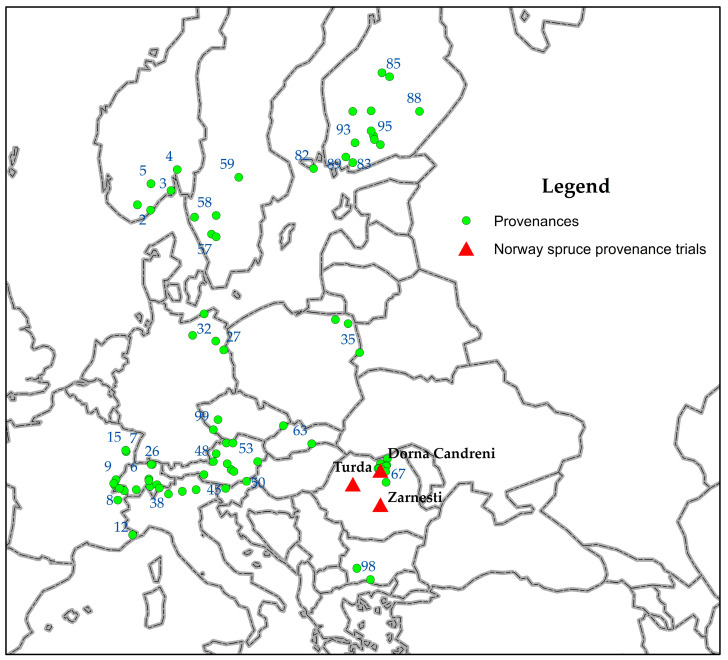
The locations of the Norway spruce provenances (dots and numbers) and of the provenance trials (red triangles).

The three provenance trials from Romania, Dorna Candrenilor, Zarnesti, and Turda, analysed in this study, are in the Eastern, Southern, and Western Romanian Carpathians, respectively. The Zarnesti and Dorna Candrenilor provenance trials are in the mixed beech and coniferous species zone, while Turda is in the Norway spruce vegetation zone. The climate conditions are continental, with Scandinavian–Baltic influences in the north (Dorna Candrenilor) and temperate–continental with oceanic influence in the west (Turda) and central parts (Zarnesti) [42]. The three Norway spruce provenance trials were established in 1972 [43]. Eighty-one provenances were tested in these trials, ten from Romania and the rest from twelve other European countries (Figure 1). The geographic coordinates and details about the tested provenances are presented in Supplementary Table S1. 

Provenances were planted in a randomised complete block design, each plot with 16 (4 × 4) individuals per provenance at a 2 × 2 m spacing and three blocks. No thinning or artificial pruning was performed in these provenance trials.

The wood cores were extracted in the fall of 2020. Given the advanced age of the provenance trials, where possible, four trees were randomly selected for each provenance in each repetition. With the Haglöf increment borer (Haglöf Sweden AB, Långsele, Sweden), one core/tree was extracted at breast height, on the slope line, to avoid compression and tension wood. Only one core from each tree was extracted to avoid tree damage.

The cores were then dried and progressively sanded to obtain a smooth surface. For the image to be of good quality (1200 dpi), an Epson Expression 12000XL scanner (Seiko Epson Corporation, Nagano, Japan) was used. Ring width (RW), earlywood (EW), and latewood (LW) were measured with CooRecorder software version 7.4 [44]. The latewood percentage (LWP) was calculated as the ratio between LW and RW. For each trial, the series were checked and cross-dated using the detrendeR version 1.0.5 [45] and dplR version 1.7.3 [46] packages in the R free software environment version 4.0.0 [47]. Dendrochronological series with intercorrelation values below 0.328 were excluded from the analysis. The intercorrelation is the correlation coefficient between different cores. The final number of wood cores was 2709: 947 for Dorna Candrenilor, 906 for Zarnesti, and 856 for the Turda trial.

The spline method has been used to detrend the ring series, where the frequency response is 0.50 at a wavelength of 0.67 × “series length in years”. By detrending, the tree’s natural biological growth trend is estimated and removed, to produce the dimensionless ring width index (RWI). 

Climatic data for the period 1972–2020 were obtained using the Climate Downscaling Tool (ClimateDT) [48], at a resolution of 1 km. For the period 1961–1990, it uses a high-resolution grid as the baseline, while for the historical period, it uses monthly anomalies derived from Climatic Research Unit gridded Time Series (CRU-TS) [49]. For future scenarios, UKCP18 surfaces [50] are being used. Except for the minimum and maximum monthly temperatures, all other climatic variables were used to evaluate the relation between climate and growth.

To determine the years with meteorological droughts, the Standardised Precipitation Evaporation Index (SPEI) [51] was calculated based on precipitation and the potential evapotranspiration (PET) for the 1972–2020 period, using the SPEI R package version 1.7 [52]. The Thornthwaite equation [53] was used for calculating PET, which can be computed only with temperature data. Using the SPEI at a time scale of 3 months (SPEI-3) (which uses the moving average of precipitation for three months, e.g., the value of SPEI-3 for March is calculated based on the precipitation and PET from January, February, and March), the drought years that recorded values below −1 were classified as follows: from −1 to −1.49, moderate drought; from −1.5 to −1.99, severe drought; less than −2, extreme drought. 

The response to drought events was evaluated using the tree resilience indices proposed by Lloret et al.: resistance (Res), recovery (Rec), resilience (Rsl), and relative resilience (relRsl) [54]. Resistance indicates the reversal of the decrease in radial growth during the drought. It is the ratio of RW during the drought (Dr) and before the drought (preDr). Values above 1 indicate high tolerance, while values below 1 mean low tolerance. Recovery indicates the capacity to revitalise after a drought. It is the ratio of RW after drought (postDR) and RW during drought. Resilience indicates the capability of reaching a pre-drought RW after a drought event. It is the ratio of RW after drought and RW before the drought. Values below 1 indicate long-term growth reductions. Relative resilience was calculated using the formula relRsl = (postDr − Dr)/preDr. It is the resilience accounting for the impact that the drought year had. The before and after RW (preDR and post DR) were calculated as average values for a period of three years before and after a year with a drought event.

To verify that the years with extreme drought events that we identified were actually years when the trees were stressed, pointer years were calculated in the dplR R package version 1.7.3 [46], based on the Becker algorithm [55]. The term ‘pointer years’ denote years in which the greater part of the trees show very narrow (negative pointer year) or wide ring widths (positive pointer year) [56,57]. A threshold of 60% of influenced trees was used to identify the pointer years.

### 2.2. Statistical Analysis

An analysis of the genetic variation in wood traits and tree resilience indices was performed for each trial site, considering that droughts and post-drought effects vary between ecoregions [58]. Using the lmerTest R package version 3.1-3 [59], a mixed linear model was applied and tested, where provenance and year x provenance were considered random effects and the block as a fixed effect.

Xijk = μ + Pj + Bk + Yl + Pj × Yl + ejklm,(1)

where μ is the general mean, Pj is the effect of the jth provenance, Bk is the effect of the kth block, Yl is the effect of the lth year, Pj × Yl is the provenance-by-year interaction, and ejklm is the error term associated with jklm trees [60].

Pearson coefficients were used to assess the correlations between the geographical coordinates (latitude, longitude, altitude) of the provenances’ origin and the wood traits and mean tree resilience indices, to examine the degree to which trait variation is influenced by local adaptation to climatic conditions from the origin location. The coordinates were not transformed prior to the correlation analyses.

The response and correlation function analysis from treeclim R package version 2.0.6.0 [61] was used to assess the non-stationarity of climate–growth correlations in each provenance trial. Non-stationarity was defined as a modification in the relationship’s magnitude or direction between variables over time [62]. The climatic variables used for this analysis were monthly values of temperature and precipitation from each year, from March to September (growing season), for the 1981–2020 interval. Subperiods of 25 years, each following one starting a year later, were analysed, resulting in 16 subperiods.

## 3. Results

### 3.1. Drought Years Identification

**Figure 2:**
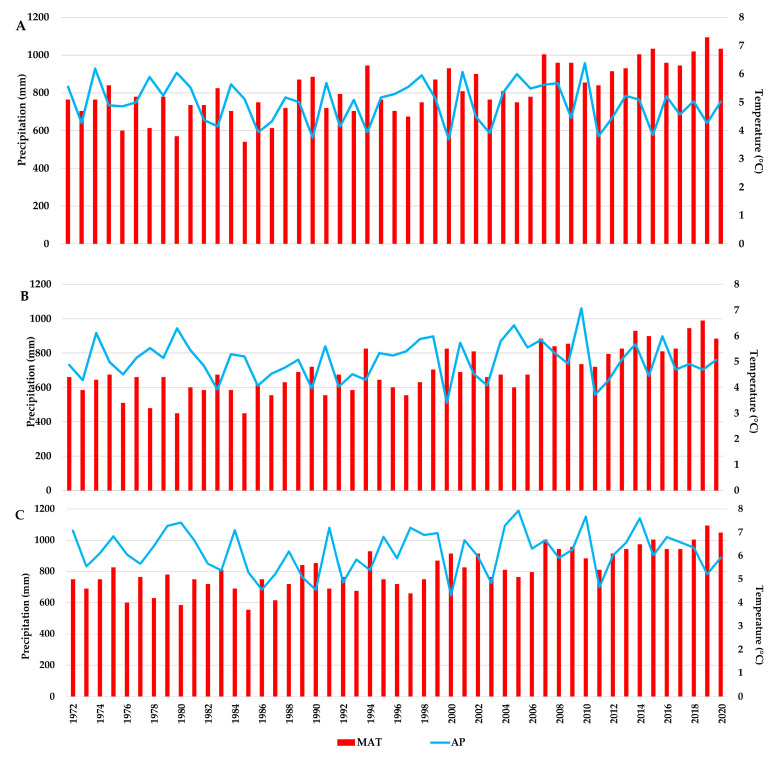
The variation in mean annual temperature (MAT) and annual precipitations (AP) for the Norway spruce provenance trials for the 1972–2020 period; (**A**)—Dorna Candrenilor, (**B**)—Turda, (**C**)—Zarnesti.

In each study site, large variations in annual mean temperature and annual precipitation amounts were recorded (Figure 2). The year 2000 had the lowest amount of precipitation for the analysed period in all three sites, ranging from 511 mm at the Turda trial, to 646 mm at the Zarnesti trial. The highest MAT was recorded for the year 2019, in all three trials, with values ranging from 6.6 °C at the Turda trial to 7.3 °C at the Dorna Candrenilor and Zarnesti trials, respectively.

Large variations in temperature and precipitation can be seen for the analysed period (1972–2020). For temperature, both the annual mean and the one from the warmest quarter, there is a clear trend of increasing values. Regarding precipitation, although the annual amount has decreased in the last decade, the most remarkable fact is the difference between 2010 and the 2011 APs. The year 2010 was the one with the highest amount of annual precipitation for the Dorna Candrenilor and Turda trials and the second highest for the Zarnesti one. In contrast, the year 2011, even though it was not classified as extreme according to SPEI-3 in our study, has one of the lowest APs from the analysed period: the second, third, and fourth ones for the Turda, Dorna Candrenilor, and Zarnesti trials, respectively.

The year 2000 had the lowest AP for the analysed period, the values dropping to 511, 553, and 646 mm in the Turda, Dorna Candrenilor, and Zarnesti trials, respectively. The optimum climate for Norway spruce in Romania is represented by an MAT between +4 and +7 °C and an AP between 800 and 1200 mm [63]. At Dorna Candrenilor, the AP dropped in seven years even below the limit value of 600 mm (1986, 1990, 1994, 2000, 2003, 2011, 2015) and only 16 out of the 49 years had optimum AP values (above 800 mm). At the Turda trial, regarding AP, four years had values below the limit value. At the Zarnesti trial, ten years showed APs below the optimum value, but none were below the limit. 

**Figure 3:**
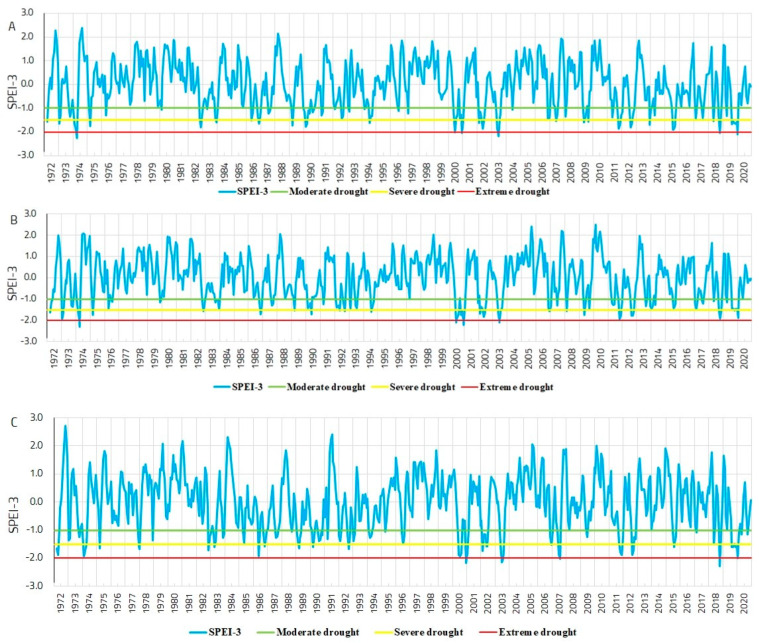
Variation in SPEI-3 for the Norway spruce provenance trials, for the period 1972–2020; (**A**)—Dorna Candrenilor, (**B**)—Turda, (**C**)—Zarnesti.

For the period 1972–2020, moderate, severe, and extreme drought years were identified based on the SPEI-3 values (Figure 3). In the Dorna Candrenilor and Turda trials, 23 years with severe and extreme droughts have been identified for the analysed period, while for Zarnesti, the number was 24 (Table 1).

**Figure 4:**
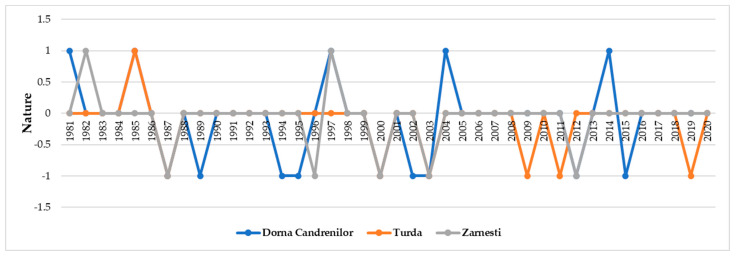
The pointer years in each Norway spruce provenance trial. A nature value of 1 means the year had a positive influence on the trees’ ring growth, while a value of −1 means the year had a negative influence on the trees’ ring growth.

The number of years with extreme drought varied among the sites and ranged between three at the Turda trial to five at the Dorna Candrenilor trial. The years when extreme drought occurred in all three provenance trials were 2000 and 2003, as confirmed by the pointer years analysis (Figure 4).

### 3.2. Norway Spruce Series 

For the analysed period, the mean RW ranged from 2.55 mm, at the Zarnesti trial, to 2.81 mm, at the Turda trial.

The mean series intercorrelation (rbar) was calculated for each provenance trial. It is the mean correlation coefficient among the tree-ring series, and it ranged from 0.876 to 0.908 (Table 2).

### 3.3. The Effect of Provenance and Year on Radial Width and Latewood Percentage

In each provenance trial, there were significant differences between provenances and years regarding wood width and LWP (Table 3). The block effect was significant for all traits, except RW at the Turda trial. Regarding year x provenance interaction, it was significant for RW and EW at the Zarnesti trial, and for RW, EW, and LWP at the Dorna Candrenilor trial. 

**Figure 5:**
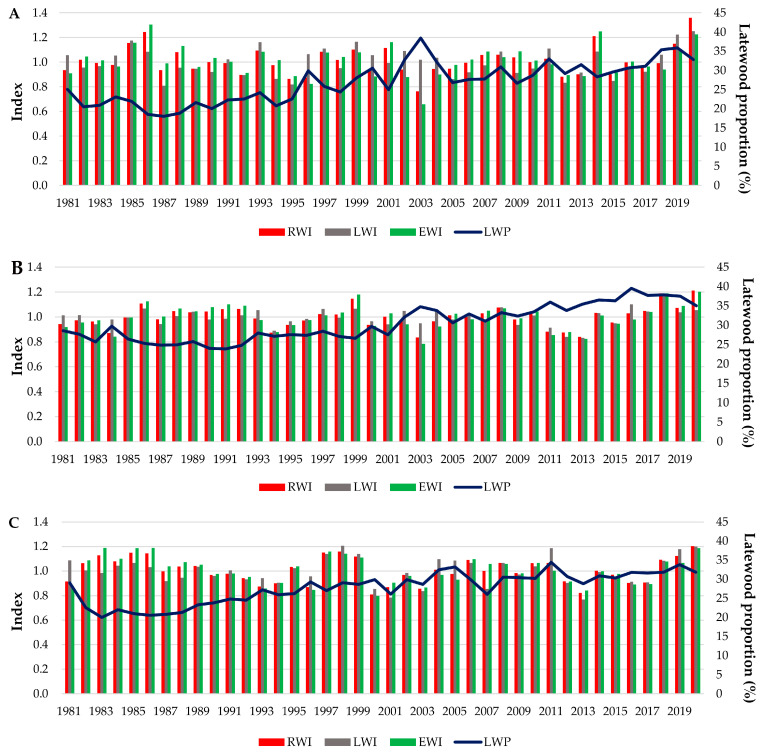
Variation in the wood traits of Norway spruce over the years for the period 1981–2020; (**A**)—Dorna Candrenilor, (**B**)—Turda, (**C**)—Zarnesti. RWI –ring width index; EWI –earlywood width index; LWI—latewood width index; LWP—latewood percentage.

The wood trait variation over the years for each provenance trial can be seen in Figure 5. 

### 3.4. Genetic Variation in Drought Response

**Figure 6:**
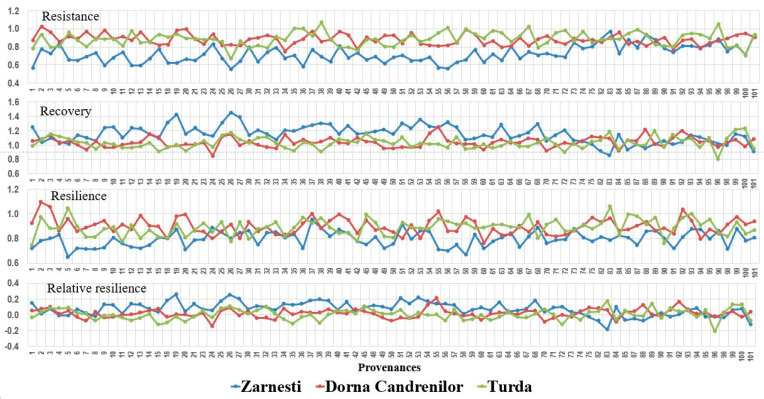
Variation in tree resilience indices of Norway spruce provenances for the year 2000.

Analysing the provenances’ different responses to the drought from the year 2000 (Figure 6), significant differences were found between the provenances for all tree resilience indices in the Zarnesti trial. At the Turda trial, the differences were significant for all indices, except resilience, while at Dorna Candrenilor, resilience was the only factor with significant differences between provenances (Table 4). 

The best results regarding resistance in the year 2000 were obtained at the Turda and Dorna Candrenilor provenance trials, with a mean resistance of 0.89 and 0.88, respectively, higher by 23.61 and 22.22%, respectively, than the mean resistance from the Zarnesti provenance trial (0.72).

The mean value for recovery of the year 2000 was above one in all three provenance trials, the highest being achieved in the Zarnesti trial, 1.15 ± 0.12 (SD).

The provenance trials Dorna Candrenilor and Turda had the greatest mean resilience for the year 2000, 0.90 and 0.89, respectively. 

The mean of the relative resilience was between 0.0, in the Turda trial, and 0.07, in the Zarnesti trial.

For the drought event from 2003, significant differences between provenances for all tree resilience indices were found only in the Zarnesti trial. No significant differences between provenances were found for any tree resilience indices in the other two sites (Table 4).

The drought event from 2000 impacted the three provenance trials differently. Its highest impact was at the Zarnesti trial, where no Norway spruce provenance had a mean resistance above 1; in other words, all provenances were affected (Figure 6). 

The highest amplitude for the mean value of the provenances’ resistance was at the Zarnesti trial, ranging from 0.55, provenance 26-Winterthur, to 0.97, provenance 83-Bramarv. For this trial, the site mean was 0.72, the lowest of the three trial sites. For the Turda trial, it ranged from 0.67, provenance 26-Winterthur to 1.07, provenance 38-Val Di Fiemme. And for the Dorna Candrenilor trial, the mean provenance resistance ranged from 0.75, provenance 34-Borki, to 1.02, provenance 2-Branstad.

Only five provenances had values below 1 regarding recovery at the Zarnesti trial: 88-Pualanka, 85-Heinola, 82-Sund, 101-Valke Karlovice, and 83-Bramarv. At the Dorna Candrenilor trial, the number was 20 provenances, and at the Turda trial, it was 28.

No provenances had a mean resilience above one at the Zarnesti trial, with values ranging from 0.65, provenance 5-Seljord, to 0.95, provenance 37-Latemar. The Northern provenances 2-Branstad, 3-Sandar, 92-Padasjoki, and 55-Munkahus were the ones that had a resilience above 1 at the Dorna Candrenilor trial, for the year 2000; 37-Latemar, 40-Wietersdf and 20-Le Brassus had a mean resilience of 1; the rest had mean values ranging from 0.76, provenance 60-Keletbukki Allami, to 0.99, provenance 20-Le Brassus. At the Turda trial, values above 1 for resilience were shown by provenances 83-Bramarv (1.06) and 5-Seljord (1.05). Provenances 93-Urjala and 85-Heinola had a resilience of 1, and the rest had values ranging from 0.75, provenance 1-Senum, to 0.99, provenance 45-Hollenburg and 67-Frasin.

### 3.5. Phenotypic Correlations

Significant and positive correlations between elevation and wood traits were found only at the Dorna Candrenilor trial, except LWP (Table 5). Also, at this trial, the longitude was negatively and significantly correlated with LW and LWP. At the Turda trial, longitude was negatively correlated with EW and positively correlated with LWP. 

Regarding latitude, significant and negative correlations were found at the Dorna Candrenilor trial for all traits except LWP. At the Zarnesti trial, latitude was negatively correlated with LWP only. No significant correlations were found between wood traits and latitude at the Turda trial.

The correlation between EW and LW ranged from 0.309 at Dorna Candrenilor to 0.753 at Turda. Correlations between RW and EW were between 0.922 in the Dorna Candrenilor trial and 0.983 in the Turda trial. Negative correlations were found between RW and LWP in the Dorna Candrenilor and Zarnesti trials. 

Regarding the correlations between RW and LW, the highest value was obtained at the Turda trial, 0.861. 

Regarding the mean of tree resilience indices from 2000 and 2003, at the Dorna Candrenilor trial, elevation was positively correlated with resistance (r = 0.128) and negatively correlated with recovery (r = −0.132) (Table 6). Latitude was positively correlated with recovery.

At the Zarnesti trial, however, no significant correlations were found between elevation and tree resilience indices. Latitude was negatively correlated with all tree resilience indices. The same for longitude, except the correlation with resistance was not significant (r = 0.120, *p* = 0.061).

At the Turda trial, no significant correlations were found regarding elevation and latitude. Longitude was positively correlated with resistance (r = 0.167), and negatively correlated with recovery (−0.151) and relative resilience (r = −0.152).

Regarding the correlations between the mean of the 2000 and 2003 tree resilience indices and mean wood traits (Table 7), resistance was negatively and significantly correlated with EW and RW at the Dorna Candrenilor trial, and with EW, LW, and RW at the Turda trial. In the same two trials, the correlations were positive with LWP.

There was a positive correlation between recovery and EW and a negative one with LWP at the Dorna Candrenilor trial. At the Zarnesti trial, there was a low correlation between recovery and LW (r = 137*). In contrast, recovery was highly correlated with EW, LW, and RW at the Turda trial.

### 3.6. The Response and Correlation Function Analysis

**Figure 7:**
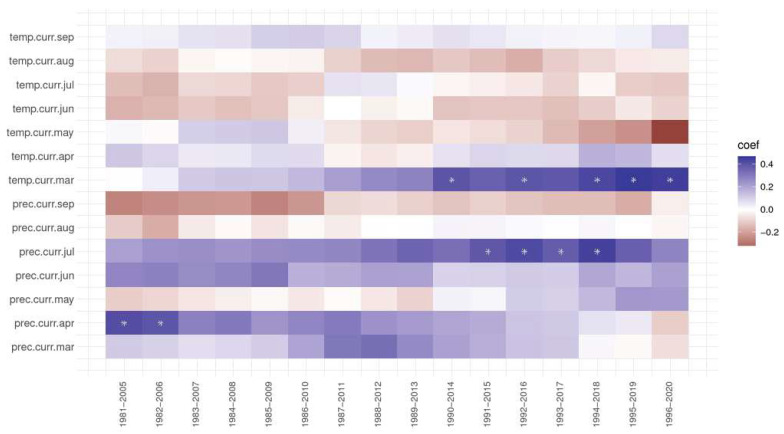
The response and correlation function for the Dorna Candrenilor trial of Norway spruce provenances. * significant at 5%.

Analysing the response and correlation function, precipitation in July always had a positive effect on ring width index at the Dorna Candrenilor trial (Figure 7), but it became significant after 1991. Contrarily, the effect of the precipitation from April slowly decreased, having even negative values for the last period, 1996–2020. The temperature of March also had a positive effect, more so after 1990. The temperature of May, although positive in the first periods, from 1981–2010, had only a negative impact later on, and it seems it is only getting stronger. 

**Figure 8:**
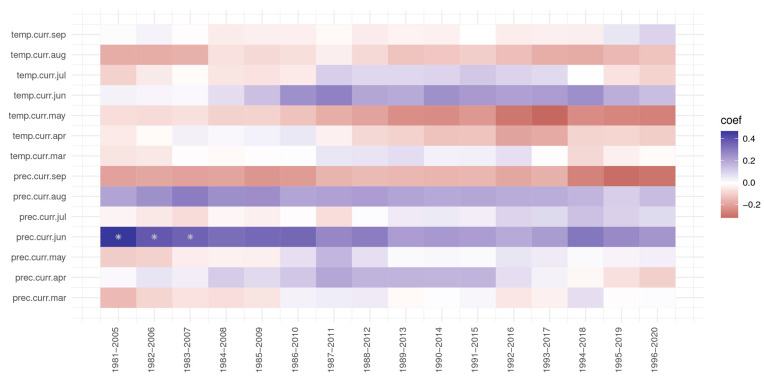
The response and correlation function for the Zarnesti trial of Norway spruce provenances. * significant at 5%.

At the Zarnesti trial, precipitation in June was significant for the first three periods of 1981–2007 (Figure 8); the value of the correlation coefficient, while still positive, was decreasing. Precipitation from September and the temperature in May, while not significant, had a negative impact but became stronger in the last periods (three and four, respectively). 

**Figure 9:**
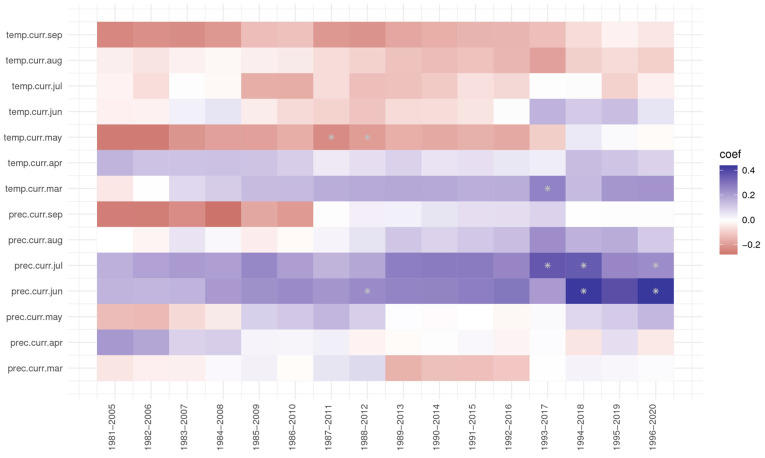
The response and correlation function for the Turda trial of Norway spruce provenances. * significant at 5%.

At the Turda trial, precipitation in June and July always had a positive effect on ring growth, but more so for the last three and four periods, respectively (Figure 9). The negative effect of March temperature for the first period was analysed, 1981–2005; it became positive starting with the 1983–2007 period, even being significant in the 1993–2017 period. The temperature of May, which was significant for the 1987–2011 and 1988–2012 periods, had a negative effect on ring growth for all the analysed periods, except the last three (1994–2020), when the correlation coefficients had values close to 0.

## 4. Discussion

The radial width and early- and latewood, as well as latewood percentage and the response to drought, of 81 Norway spruce provenances have been analysed in three long-term provenance trials located in three different geographic regions of the Romanian Carpathian Mountains.

The plant response to drought stress is very intricate, and various strategies can be adopted to endure drought events. Baldi and La Porta classify these strategies into three main groups: drought avoidance, drought resistance, and drought resilience [64]. The drought avoidance strategy (e.g., deep roots or stomatal adjustments) represents a tree’s capacity to minimise the amount of water lost and maximise water uptake [65,66]. Drought resistance was defined as the capacity of a tree to endure stress, and drought resilience was defined as how swiftly a tree can resume normal growth, once the stress stopped [54,67]. The traits and processes that are involved in the drought avoidance, resistance, and resilience of conifers have been reviewed in other studies [68].

The negative effect of rising temperatures on conifer growth was also observed in the study of Gómez-Aparicio et al. [69], which can be connected to a higher atmospheric water demand, which causes greater drought stress. Positive correlations between Norway spruce RW and the climate of July and August of the current year but also October of the previous year, and a negative effect of the temperature of September from the previous year, have been reported in the Czech Republic [70].

The precipitation amounts from June and July also became important for pine growth in Lithuania [71], a change from earlier studies [72]. In the study of Lévesque [16], soil water availability from December to July was also important. Roibu [73] found that the correlations with temperature and precipitation also changed over time for ash and oak from the Republic of Moldova, using moving window correlations of 25 years.

The differences between provenances regarding wood traits and drought response were very significant in each trial. This can be explained by the broad geographic amplitude of the tested provenances, which comprised provenances of the Alpine range, the Bohemian Massive, the Carpathian Mountains, the Rila and Rhodope Mountains, the Vosges and Jura Mountains, and provenances from Northern Europe (Sweden, Norway, and Finland), as well as provenances from NE Germany and NE Poland. The high adaptive variation of Norway spruce has been observed in Central and Southeastern Europe [32].

Recovery was positively correlated with latitude in Dorna Candrenilor but negatively correlated with it in the Zarnesti trial. In the Dorna Candrenilor trial, which is a forest site of superior quality, the Southern provenances had a greater resistance and thus a lower recovery. On the other hand, in the Zarnesti trial, which is a forest site of medium–inferior quality, the Southern provenances had a lower resistance and, thus, a greater recovery (see Figure 6).

The provenances with a high radial growth and good resistance and/or recovery in all three trials are the following: 53-Neustift, 49-Redl-Zipf-Fuchsberg, and 40-Wietersdf, from the Eastern Alps; 19-Kerns and 25-Wassen, from the Central Alps; 31-Bremenhagen, from NE Germany; 99-Zelesna Ruda, from the Bohemian Forest Mountains (Czech Republic); and 67-Frasin, 70-Coşna, 71-Moldovița, and 75-Broşteni, from the Eastern Romanian Carpathians.

Provenance 53-Neustift, from the Eastern Alps, had a great mean RW in all three trials. The resistance for the year 2000 had values between 0.65, at the Zarnesti trial, to 0.86, at the Turda trial. Regarding recovery, it ranged between 0.97 and 1.35 at the Dorna Candrenilor and Zarnesti trials, respectively. And the resilience was between 0.80 and 0.89 at the Dorna Candrenilor and Turda trials, respectively.

Another valuable provenance is 25-Wassen, from the Central Alps; with a mean RW of 3.11 and 3.52 mm at the Dorna Candrenilor and Turda trial, respectively, it was the first and third ranking in these provenance trials. In the Zarnesti trial, it occupied rank 32, with a mean RW of 2.58 mm. It was not very resistant, with values for the resistance index ranging from 0.66, at the Zarnesti trial, to 0.85, at the Turda trial. The resilience had values between 0.86 and 0.93 at the Zarnesti and Turda trial, respectively. This provenance’s recovery had great values: from 1.12 to 1.38 at the Dorna Candrenilor and Zarnesti trials, respectively

Provenance 19-Kerns, again from the Central Alps, presented good growth in all three trials and a good recovery (from 0.99 to 1.42 in the Dorna Candrenilor and Zarnesti trials, respectively). It had a great resistance at the Dorna Candrenilor trial (0.98), but less so at the Turda and Zarnesti trials, 0.86 and 0.62, respectively.

Romanian provenance 75-Broşteni had a mean RW above average (2.92) at Dorna Candrenilor and Turda trials, and below average (2.46) at the Zarnesti trial. The resistance was between 0.81 and 0.88, at the Zarnesti and Dorna Candrenilor trials, respectively. Even though the resistance was low, it was above average at the Zarnesti trial, but below average in the other two trials. This provenance managed to obtain a recovery above one in all three trials, and a resilience close to one, 0.97 in the Dorna Candrenilor trial. In the other two trials, it was below average, but close to it, 0.88 compared with 0.89, and 0.77 compared with 0.80, in Turda and Zarnesti trial, respectively.

Provenance 70-Coşna, likewise from Romania, had a great RW in all three trials too. It had a high resistance in the Dorna Candrenilor trial, 0.92, but lower and below average in the Turda trial, 0.85, and lower but above average in the Zarnesti trial, 0.73. In contrast, the recovery of this provenance was high in the Turda and Zarnesti trial, but below one in the Dorna Candrenilor trial (0.92). The resilience was close to the mean value of the trials, but higher only at the Turda trial.

It should be noted that the extreme drought from 2003 and the severe drought from 2002 affected the value of the calculated recovery, resilience, and relative resilience for the extreme event from 2000. Also, the values of resistance, resilience, and relative resilience of the year 2003 were influenced by the extreme event from 2000 and the severe drought from 2002. Further studies should investigate extreme events that are more distant, a fact that is becoming difficult, considering recent events from the 2018–2022 period in Europe [74], or the projections for Romania [75].

Some provenances may represent artificial stands of unknown origin, as is clearly the case for the provenances from Hungary and Northeastern Germany, which are outside the range. Molecular analyses should be conducted in future research to confirm the origin of thsee provenances, especially for the ones selected for being the most resilient. It is important to know the exact origin of the valuable provenances, so that they can be used in breeding programs and assisted migration and for the conservation of the species’ genetic resources.

## 5. Conclusions

The number of years with extreme drought events has increased in the last two decades in the Romanian Carpathian Mountains. We found high genetic variation in the wood characteristics and drought response of the Norway spruce provenances tested in this study. These results could be further exploited in breeding, seed selection, and genetic conservation programs but also represent a stage in the development of the adaptation strategy of the species.

The drought year and the environmental conditions of the planting site influenced the provenances’ drought response. Norway spruce provenances with high growth rates are the most affected by extreme droughts, a fact highlighted by the negative correlations between RW and resistance in all three trials. Moreover, a prolonged period for recovery was needed after the drought, indicating a lower fitness for drought tolerance of Norway spruce. However, several provenances with high radial growth and good resistance and/or recovery have been identified. 

Provenances that performed better in common garden experiments could be used in assisted migration, even in the proximity of the current natural range. The adaptation process could be assisted by relying on assisted migration, especially at the edges of the species distribution, and the genetic diversity would be easier to conserve and increase. 

## Figures and Tables

**Table 1:** Years and months with severe and extreme drought events for each Norway spruce trial. The bold represents the extreme events, based on SPEI-3.

Year	Months with Severe and Extreme Droughts		
	Dorna Candrenilor	Turda	Zarnesti
1972	March	March	March, April
1973	Jan	January, February	-
1974	Febr.–**April**	Febr.–**April**	Febr.–April
1975	March	March	March
1977	-	-	December
1978	-	-	January
1982	November	November	November
1983	December	December	April
1986	May, November	November	May, October
1989	March	March	March
1990	Febr., March	May	March
1992	-	May, September	September
1994	July	July	-
1996	-	-	July
2000	**June, December**	**June**–August, October–**December**	June, July, August, **December**
2001	January	-	January
2002	Febr., May, June	Febr., May, June	febr., May, June
2003	May, **June**	May, **June**	May, **June**, **July**, August
2006	-	December	December
2007	June	-	June, **July**
2008	-	February	-
2009	May, September	May	-
2011	Oct., November	October, November	October, November
2012	Aug, September	August–October	August, September
2013	December	-	-
2015	July, August, September	-	July
2017	-	February	-
2018	May, **October**	September–November	May, **October**
2019	August-December	October	August, September, November, December
2020	**January**	January	January

**Table 2:** Descriptive statistics of the Norway spruce series at 49 years after planting.

	rbar	Provenance rbar min	Provenance rbar max	Mean RW (mm)	Min RW (mm)	Max RW (mm)
Dorna Candrenilor	0.908	0.802, prov 90	0.961, prov 54	2.73	2.15, prov 83	3.11, prov 25
Turda	0.876	0.729, prov 67	0.941, prov 75	2.81	2.31, prov 83	3.92, prov 10
Zarnesti	0.876	0.705, prov 18	0.942, prov 42	2.55	2.13, prov 94	3.18, prov 55

rbar—the mean series intercorrelation; RW—ring width.

**Table 3:** The results for the random and fixed effects of the wood width and LWP in each Norway spruce provenance trial evaluated at age 49.

	Trait	LRTp	LRT Year x prov	MS B	MS Year	Mean ± SD
Zarnesti	RW	711.55 ***	66.98 ***	91.56 ***	1116.66 ***	2.55 ± 0.19
	EW	677.85 ***	84.63 ***	38.94 ***	739.12 ***	1.90 ± 0.17
	LW	376.41 ***	0.00	11.12 ***	46.88 ***	0.65 ± 0.05
	LWP	510.66 ***	0.00	4583.1 ***	13,944.9 ***	27.78 ± 1.65
Dorna Candrenilor	RW	712.85 ***	12.42 ***	47.88 ***	1996.86 ***	2.73 ± 0.18
	EW	579.62 ***	30.60 ***	12.07 ***	1310.91 ***	2.08 ± 0.14
	LW	639.68 ***	1.29	16.981 ***	65.855 ***	0.66 ± 0.07
	LWP	491.92 ***	26.37 ***	3207.5 ***	20,586.8 ***	27.06 ± 1.54
Turda	RW	1259.9 ***	0.00	2.09	1315.16 ***	2.81 ± 0.29
	EW	1003.0 ***	0.00	2.28 *	835.49 ***	2.03 ± 0.22
	LW	996.03 ***	0.00	0.66 **	56.16 ***	0.78 ± 0.08
	LWP	326.7 ***	0.00	1389.3 ***	15,916 ***	30.76 ± 1.62

*, **, *** Significant at 5%, 1%, and 0.1%, respectively; LRTp—likelihood ratio test for provenance random effect; LRT year x prov—likelihood ratio test for provenance x year random effect; MS B—mean squares for block effect; MS Year—mean squares for year effect; RW—ring width; EW—earlywood width; LW—latewood width; LWP—latewood percentage.

**Table 4:** The results for the random and fixed effects of the tree resilience indices in each Norway spruce provenance trial evaluated in their common extreme years.

Year	Provenance Trial	Trait	LRTp	MS B
2000	Dorna Candrenilor	Resistance	0.084 ns	0.118 *
		Recovery	2.593 ns	0.011 ns
		Resilience	5.418 *	0.063 ns
		Rel. resilience	1.966 ns	0.009 ns
	Turda	Resistance	7.859 **	0.124 *
		Recovery	8.014 **	0.0009 ns
		Resilience	1.850 ns	0.120 *
		Rel. resilience	9.308 **	0.0002 ns
	Zarnesti	Resistance	25.268 ***	0.254 ***
		Recovery	38.035 ***	0.666 ***
		Resilience	7.015 **	0.048 ns
		Rel. resilience	32.521 ***	0.458 ***
2003	Dorna Candrenilor	Resistance	2.604 ns	0.192 ***
		Recovery	0 ns	0.073 ns
		Resilience	0.429 ns	0.234 ***
		Rel. resilience	5.12 × 10^−13^ ns	0.029 ns
	Turda	Resistance	2.297 ns	0.087 ns
		Recovery	0.021 ns	0.371 *
		Resilience	0.879 ns	0.123 *
		Rel. resilience	1.42 × 10^−12^ ns	0.308 **
	Zarnesti	Resistance	14.843 ***	0.219 **
		Recovery	24.834 ***	0.576 ***
		Resilience	14.084 ***	0.064 ns
		Rel. resilience	22.538 ***	0.383 **

*, **, *** Significant at 5%, 1%, and 0.1%, respectively; ns—non-significant; LRTp—likelihood ratio test for provenance random effect; MS B—mean squares for block effect.

**Table 5:** Correlation coefficients between wood traits and geographic coordinates of Norway spruce provenances at 49 years after planting.

Provenance Trial		LW	LWP	RW	Latitude	Longitude	Elevation
Dorna Candrenilor	EW	0.309 ***	−0.445 ***	0.922 ***	−0.185 **	−0.043	0.179 ***
	LW		0.625 ***	0.653 ***	−0.159 *	−0.162 *	0.220 **
	LWP			−0.099	−0.005	−0.135 *	0.034
	RW				−0.212 *	−0.100	0.232 *
Zarnesti	EW	0.452 ***	−0.468 ***	0.963 ***	0.048	−0.010	−0.065
	LW		0.502 ***	0.676 ***	−0.048	−0.042	−0.016
	LWP			−0.235 **	−0.131 *	−0.020	0.057
	RW				0.025	−0.021	−0.059
Turda	EW	0.753 ***	−0.468 ***	0.983 ***	−0.068	−0.130 *	0.010
	LW		0.134 *	0.861 ***	−0.036	−0.058	0.065
	LWP			−0.324 ***	0.070	0.155 *	0.029
	RW				−0.062	−0.117	0.026

*, **, *** Significant at 5%, 1%, and 0.1%, respectively; RW—ring width; EW—earlywood width; LW—latewood width; LWP—latewood percentage.

**Table 6:** Correlation coefficients between mean tree resilience indices and geographic coordinates of Norway spruce provenances at 49 years after planting.

Provenance Trial		Latitude	Longi-Tude	Elevation
Dorna Candrenilor	Resistance	−0.037	−0.114	0.128 *
	Recovery	0.131 *	0.038	−0.132 *
	Resilience	0.074	−0.099	0.043
	Rel. resilience	0.123	0.025	−0.103
Zarnesti	Resistance	0.215 ***	0.120	−0.121
	Recovery	−0.236 ***	−0.317 ***	0.111
	Resilience	−0.223 ***	−0.277 ***	0.124
	Rel. resilience	−0.239 ***	−0.312 ***	0.117
Turda	Resistance	0.103	0.167 **	0.047
	Recovery	−0.099	−0.151 *	0.048
	Resilience	0.078	0.019	0.049
	Rel. resilience	−0.047	−0.152 *	−0.012

*, **, *** Significant at 5%, 1%, and 0.1%, respectively.

**Table 7:** Correlation coefficients between mean tree resilience indices and wood traits of Norway spruce provenances at 49 years after planting.

Provenance Trial		Resistance	Recovery	Resilience	Rel. Resilience
Dorna Candrenilor	EW	−0.255 ***	0.153 *	−0.175 **	0.106
	LW	0.108	−0.097	0.018	−0.106
	LWP	0.289 ***	−0.207 **	0.150 *	−0.173 **
	RW	−0.159 *	0.082	−0.132 *	0.042
Zarnesti	EW	−0.007	0.063	−0.043	0.077
	LW	−0.035	0.137 *	0.084	0.141 *
	LWP	−0.035	0.052	0.089	0.034
	RW	−0.016	0.094	−0.011	0.106
Turda	EW	−0.370 ***	0.347 ***	−0.050	0.330 ***
	LW	−0.267 ***	0.277 ***	−0.004	0.260 ***
	LWP	0.192 **	−0.065	0.141 *	−0.090
	RW	−0.360 ***	0.345 ***	−0.039	0.327 ***

*, **, *** Significant at 5%, 1%, and 0.1%, respectively; RW—ring width; EW—earlywood width; LW—latewood width; LWP—latewood percentage.

## Data Availability

Data presented in this study are available from the corresponding author upon request.

## Supplementary Materials

The following supporting information can be downloaded at: https://www.mdpi.com/article/10.3390/plants13162172/s1, Table S1: Details about the tested Norway spruce provenances and the provenance trials. Table S2: Details about the Norway spruce provenance trials.
