# Supplementary material for: Tree Resilience Indices of Norway Spruce Provenances Tested in Long-Term Common Garden Experiments in the Romanian Carpathians
Source: Plants (Basel). 2024 Aug 6;13(16):2172. doi: 10.3390/plants13162172 (PMC11360809; doi:10.3390/plants13162172)
Supplement: Supplementary file 1 [file plants-13-02172-s001.zip › plants-3080563-supplementary.pdf]

Table S1. Details about the tested Norway spruce provenances

MAT-mean annual temperature (°C), MAP-mean annual precipitations (mm), for 1901-1970.

Number of provenance	Name of provenance	Country	Group	Lat. N	Long. E	Altit.	MAT	MAP
1	Senum	Norway	Northern	58.67	7.75	390	4.7	1149
2	Branstad	Norway	Northern	58.47	8.7	45	7.1	1028
3	Sandar	Norway	Northern	59.2	10.18	50	6.3	1259
4	Bagstad	Norway	Northern	59.97	10.63	160	5.3	779
5	Seljord	Norway	Northern	59.45	8.73	120	5.9	667
6	Straiture I	France	French	48.12	6.93	650	7.6	1011
7	Straiture II	France	French	48.12	6.93	650	7.6	1011
8	La Ganne	France	Western Alps	45.73	6.33	900	7.9	1262
9	Mignovillard III	France	French	46.75	6.2	1000	6.5	1285
10	Plan de Cosaques	France	French	46.48	6.1	1170	5.7	1579
11	Morzine	France	Western Alps	46.2	6.8	1750	2.6	1938
12	Lantosque	France	Western Alps	43.95	7.4	1500	5.6	1024
13	Saint Laurent II	France	French	46.57	6.03	960	6.9	1621
14	Plan Bois	France	Western Alps	46.32	6.45	500	9.8	1179
15	Gerardmer I	France	French	48.18	6.9	650	7.6	1401
18	Eptingen	Switzerland	Central Alps	46.78	8.58	1040	6.5	1221
19	Kerns	Switzerland	Central Alps	46.42	8.67	1460	4.3	1472
20	Le Brassus	Switzerland	Central Alps	46.5	9.17	1310	5.1	818
21	Lukmenier	Switzerland	Central Alps	46.33	9.33	1500	4.1	1350
23	Stampa	Switzerland	Central Alps	46.25	7.67	1600	3.6	675
24	Tagevillen	Switzerland	Central Alps	47.5	8.83	520	8.9	1075
25	Wassen	Switzerland	Central Alps	46.7	8.58	1160	5.8	1058
26	Winterthur	Switzerland	Central Alps	47.5	8.75	545	8.8	1072
27	Bodenseichen	Germany	NE Germany	52.78	14	80	8.2	487

30	Kiekindermarx	Germany	NE Germany	53.42	11.75	86	8.2	569
31	Brmenhagen	Germany	NE Germany	54.33	12.58	20	8.1	583
32	Gandenitz	Germany	NE Germany	53.17	13.42	25	8.0	546
33	Wigry	Poland	NE Poland	53.92	23	130	6.6	563
34	Borki	Poland	NE Poland	54.08	22.08	160	7.7	542
35	Bialowieza	Poland	NE Poland	52.67	23.83	150	6.8	571
36	Brajes	Italy	Eastern Alps	46.25	12	1425	4.2	1281
37	Latemar	Italy	Central Alps	46.17	11	1700	2.8	884
38	Val Di Fiemme	Italy	Central Alps	46.03	10	1350	5.0	1046
39	Klaunz Bannwald	Austria	Eastern Alps	47	12.57	1750	1.7	1138
40	Wietersdf	Austria	Eastern Alps	47.25	14.53	800	6.4	726
41	Eppenstein	Austria	Eastern Alps	47.15	14.73	965	7.0	714
42	Rotlgut Liezen	Austria	Eastern Alps	47.53	14.25	800	6.3	1000
45	Hollenburg	Austria	Eastern Alps	46.32	14.13	1125	5.2	2365
48	Strabwalchen	Austria	Eastern Alps	47.63	13.25	650	7.4	1669
49	Redl-Zipf-Fuchsberg	Austria	Eastern Alps	48.02	13.45	550	7.9	1047
50	Hoyos-Ernest-reith	Austria	Eastern Alps	46.67	15.65	530	9.5	1044
51	Herfenberg	Austria	Bohemian	48.53	14.18	750	6.3	994
52	Sandl-bei-Freistadt	Austria	Bohemian	48.53	14.67	975	5.4	770
53	Neustift	Austria	Eastern Alps	47.63	16.45	620	8.4	456
54	Kolarp	Sweden	Northern	57.55	13.12	160	6.1	777
55	Munkahus	Sweden	Northern	58.2	11.88	20	7.2	701
56	Anfasterod	Sweden	Northern	58.2	11.88	50	7.2	701
57	Mossebo	Sweden	Northern	57.45	13.45	260	5.6	827
58	Aspas	Sweden	Northern	58.27	13.45	170	6.0	539
59	Nytthan	Sweden	Northern	59.68	15.08	115	5.0	638
60	Keletbukki Allami	Hungary	Western Carpathians	48.5	20.37	300	8.7	705
61	Nyugatbukki Allami	Hungary	Western Carpathians	48.5	20.37	300	8.7	705
63	Nyugatbukki Allami	Hungary	Western Carpathians	48.5	20.37	300	8.7	705
64	Gheorghieni	Romania	Eastern (Romanian) Carpathians	46.62	25.75	1000	5.2	602

66	Marginea	Romania	Eastern (Romanian) Carpathians	47.77	25.83	670	6.4	619
67	Frasin	Romania	Eastern (Romanian) Carpathians	47.47	25.8	760	6.1	719
68	Breaza	Romania	Eastern (Romanian) Carpathians	47.53	25.33	1250	3.2	812
70	Coşna	Romania	Eastern (Romanian) Carpathians	47.3	25.17	1025	4.7	763
71	Moldoviţa	Romania	Eastern (Romanian) Carpathians	47.37	25.57	980	4.9	635
72	Dorna Candrenilor	Romania	Eastern (Romanian) Carpathians	47.28	25.25	1000	4.9	767
73	Stulpicani	Romania	Eastern (Romanian) Carpathians	47.42	25.75	980	4.8	681
74	Galu	Romania	Eastern (Romanian) Carpathians	47.25	25.42	650	7.0	701
75	Broşteni	Romania	Eastern (Romanian) Carpathians	47.15	25.72	940	5.3	627
82	Sund	Finland	Northern	60	20.5	20	5.4	508
83	Bramarv	Finland	Northern	60.22	23.33	20	4.8	606
84	Pihtipudas	Finland	Northern	63.28	25.45	165	2.4	507
85	Heinola	Finland	Northern	63.15	26	275	2.4	544
87	Kourevesi	Finland	Northern	61.17	24.83	100	3.6	586
88	Pualanka	Finland	Northern	62	28.17	140	2.8	553
89	Pielisjarvi	Finland	Northern	60.42	22.83	50	4.8	557
90	Mantta	Finland	Northern	62.02	24.67	120	3.3	557
91	Jokioinen	Finland	Northern	62	23.33	190	3.1	557
92	Padasjoki	Finland	Northern	60.85	25.33	85	3.9	573
93	Urjala	Finland	Northern	60.92	23.5	130	3.9	540
94	Janakkala	Finland	Northern	61.33	24.67	100	3.8	506
95	Tuusula	Finland	Northern	61.03	24.9	120	3.5	564
96	Rila	Bulgaria	Bulgarian	42.2	23.63	1300	6.6	881
98	Rodopi Smolian	Bulgaria	Bulgarian	41.6	24.62	2000	2.4	640
99	Zelesna Ruda	Czech Republic	Bohemian	49.17	13.25	850	5.3	1071
100	Kasperske Hory	Czech Republic	Bohemian	49.63	13.58	650	7.2	632
101	Valke Karlovice	Czech Republic	Western Carpathians	49.35	18.32	780	5.4	999

Table S2. Details about the Norway spruce provenance trials

MAT-mean annual temperature (°C), MAP-mean annual precipitations (mm), for 1972-2020.

County Forest Administration	Forest district	Area (ha)	Latitude N	Longitude E	Altitude (m.a.s.l)	MAT	MAP
Suceava	Dorna Candreni	3.40	47°12'	25°20'	1000	5.4	749
Brasov	Zarnesti	2.67	45°30'	25°20'	1025	5.4	927
Cluj	Turda	3.43	46°30'	23°45'	1250	4.6	757
